# Supplementary material for: Identifying Endogenous Proteins of Perennial Ryegrass (Lolium perenne) with Ex Vivo Antioxidant Activity
Source: Proteomes. 2025 Feb 5;13(1):8. doi: 10.3390/proteomes13010008 (PMC11843917; doi:10.3390/proteomes13010008)
Supplement: Supplementary file 1 [file proteomes-13-00008-s001.zip › proteomes-3374469-supplementary.pdf]

Supplementary materials

for

Identifying endogenous proteins of perennial ryegrass (*Lolium perenne*) with *ex vivo* antioxidant activity

Kathrine Danner Aakjær Pedersen[1,†], Line Thopholm Andersen[1,†], Mads Heiselberg[1,†], Camilla Agerskov Brigsted[1], Freja Lyngs Støvring[1], Louise Mailund Mikkelsen[1], Sofie Albrekt Hansen[1], Christian Enrico Rusbjerg-Weberskov[1], Mette Lübeck[1], Simon Gregersen Echers[1,†,*]

[1] Aalborg University, Department of Chemistry & Bioscience, Fredrik Bajers Vej 7H, DK-9220 Aalborg, Denmark
† These authors contributed equally to this work.
* Author to whom correspondence should be addressed.: sgr@bio.aau.dk

Table of contents:

Table S1: Differentially abundant proteins between crude fractions.

Table S2: BLAST analysis of lead proteins annotated as fragments in SEC fractions.

Figure S1: PCA plot triplicate analysis of initial crude fractions.

Figure S2: Volcano plot of differentially abundant proteins in pair-wise comparison of grass and pulp.

Figure S3: Volcano plot of differentially abundant proteins in pair-wise comparison of juice and pulp.

Figure S4: Heatmap representation of all 1084 identified proteins across crude fractions

Figure S5: Chromatogram and calibration curve of standards for SEC fractionation.

Figure S6: Activity of all fractions for each dilution in the DPPH radical scavenging screening.

Figure S7: Reducing (*) and non-reducing SDS-PAGE analysis of the 15 selected fractions.

Figure S8: GO-term analysis of the 108 selected proteins for investigation of known antioxidant function.

Table S1: Differentially abundant proteins across crude fraction identified from pair-wise analysis of triplicate preparations using Mass Dynamics. The table shows Uniprot AC# for the lead protein within the identified MaxQuant Protein Group, protein and gene names from Uniprot, p-value, log2 fold change (FC), adjusted p-value, and if differential abundance for the protein and conditions relies on imputed values.

Grass vs. Juice						
Uniprot AC#	Protein Name	Gene	p-value	log2 FC	Adj. P-value	Imputed
I1IYB5	Histone H3	100826679	9.37E-07	-6.73	0.0004	+
A0A0Q3FE86	Mitochondrial carnitine/acylcarnitine carrier-like protein	BRADI_3g34077v3	2.91E-04	-2.73	0.0116	+
I1HAH1	Protein-ribulosamine 3-kinase, chloroplastic (EC 2.7.1.172) (Fructosamine 3-kinase-related protein)	100846855	1.68E-03	-2.02	0.0328	+
I1I9J4	Superoxide dismutase [Cu-Zn] (EC 1.15.1.1)	100831056	1.72E-05	-1.23	0.0027	
A0A0Q3IB24	Phosphoglycolate phosphatase	100836828	1.90E-04	-1.07	0.0111	
I1J014	Remorin C-terminal domain-containing protein	100841796	6.30E-06	2.06	0.0015	
I1IKN0	Plastid lipid-associated protein/fibrillin conserved domain-containing protein	100846344	2.68E-04	2.13	0.0116	+
A0A0Q3RCW9	Rhodanese domain-containing protein	BRADI_2g57590v3	3.03E-05	2.72	0.0035	+
I1HNL9	Uncharacterized protein	100834005	3.68E-04	3.23	0.0123	+
Grass vs. Pulp						
Uniprot AC#	Protein Name	Gene	p-value	log2 FC	Adj. P-value	Imputed
I1GNA1	beta-glucosidase (EC 3.2.1.21)	100846247	9.72E-05	-3.97	0.0090	+
I1H1Z7	Large ribosomal subunit protein uL6 alpha-beta domain-containing protein	100845011	7.43E-05	-3.12	0.0086	+
A0A0Q3FE86	Mitochondrial carnitine/acylcarnitine carrier-like protein	BRADI_3g34077v3	4.32E-04	-2.99	0.0286	+
I1I9E4	Cytochrome c domain-containing protein	100822260	3.22E-04	-2.95	0.0249	+
I1HEX8	Pectinesterase (EC 3.1.1.11)	100831935	5.60E-04	-2.57	0.0289	+
I1J2I2	AB hydrolase-1 domain-containing protein	100826721	4.97E-04	-2.38	0.0288	+
I1GNA2	beta-glucosidase (EC 3.2.1.21)	100822901	3.49E-05	-1.03	0.0054	
A0A0Q3EAL0	Fibronectin type III-like domain-containing protein	100820868	7.61E-06	3.55	0.0035	+
Juice Vs. Pulp						
Uniprot AC#	Protein Name	Gene	p-value	log2 FC	Adj. P-value	Imputed
I1GUH7	Peroxidase (EC 1.11.1.7)	100828063	7.34E-05	-4.97	0.0050	+
I1GNA1	beta-glucosidase (EC 3.2.1.21)	100846247	1.48E-03	-3.81	0.0244	+

I1I9E4	Cytochrome c domain-containing protein	100822260	9.13E-05	-2.87	0.0055	+
I1HEX8	Pectinesterase (EC 3.1.1.11)	100831935	1.42E-04	-2.80	0.0074	+
A0A0Q3RCW9	Rhodanese domain-containing protein	BRADI_2g57590v3	3.11E-05	-2.63	0.0037	+
A0A0Q3HE50	Peroxidase (EC 1.11.1.7)	100839855	6.59E-04	-2.03	0.0171	+
I1GNA2	beta-glucosidase (EC 3.2.1.21)	100822901	1.48E-06	-1.91	0.0007	
I1GMG7	FAS1 domain-containing protein	100825043	6.75E-04	-1.69	0.0171	+
I1GMD6	Histone H4	BRADI_1g05980v3	1.17E-05	-1.41	0.0028	
I1GLP9	Methylenetetrahydrofolate reductase	100846793	4.33E-03	-1.18	0.0452	+
B3TN65	Photosystem II reaction center protein L (PSII-L)	psbL	2.30E-03	-1.15	0.0326	
I1HA14	glutathione transferase (EC 2.5.1.18)	100821047	6.32E-04	-1.09	0.0171	
I1IDL7	Histone H2B	100846031	2.97E-05	-1.08	0.0037	
G3DQE2	chitinase (EC 3.2.1.14)	PR3-2	4.07E-03	1.21	0.0445	
I1GZN4	Annexin	100841794	3.93E-03	1.32	0.0440	+
A0A0Q3FGU5	Aminotransferase class V domain-containing protein	BRADI_3g39750v3	3.86E-03	2.23	0.0440	+
A0A0Q3NSR7	peptidylprolyl isomerase (EC 5.2.1.8)	BRADI_1g54970v3	3.29E-04	2.29	0.0132	+

Table S2: BLAST analysis of lead proteins annotated as fragments to recalculate iBAQ with new theoretical peptides (TP_{max}) from BLAST hits.

Protein fragments	Uniprot ID(s)	BLAST hit Protein name	BLAST ID(s)	TP_{max}	MW [kDa]
homogentisate 1,2-dioxygenase (Fragment)	A0A0Q3HBY5	N/A	N/A	26	57.73
Small ribosomal subunit protein uS15 N-terminal domain-containing protein (Fragment)	A0A0Q3HNB4; I1I0I5; I1GT69	Small ribosomal subunit protein uS15 N-terminal domain-containing protein	I1I0I5	8	17.09
Glycerate dehydrogenase (Fragment)	A0A0Q3J335	Glycerate dehydrogenase	A0A8I7BAP6	22	41.56
Small ribosomal subunit protein eS1	A0A0Q3K2L3; I1H8I6; I1HAS4	Small ribosomal subunit protein eS1	I1H8I6	15	30.11
Small ribosomal subunit protein bS18c (Fragment)	A0A2K2CIY9; A0A0Q3JFN1; A8Y9A8; B3TN71; A0A0Q3GNZ2	Small ribosomal subunit protein bS18c	A8Y9A8	6	18.85
GRF-type domain-containing protein (Fragment)	A0A2K2D084	GRF-type domain-containing protein	I1ILG1	7	18.33
Uncharacterized protein (Fragment)	A0A2K2DU38	Reverse transcriptase zinc-binding domain-containing protein	A0A453EWI8	27	55.34
Glucosidase II subunit alpha (Fragment)	A0A2K2DUA7	Glucosidase II subunit alpha	A0A8R7UAH6	40	102.95
SOS1 (Fragment)	A4IF20	Cyclic nucleotide-binding domain-containing protein	A0A3B6FE53	57	131.42
Cytosolic glyceraldehyde-3-phosphate dehydrogenase (Fragment)	A6YED2; A0A0Q3PVR8; I1HXQ3	Glyceraldehyde-3-phosphate dehydrogenase	A0A0Q3PVR8	17	31.62
Elongation factor 1-alpha (Fragment)	A8ILY0; I1GMP2	Elongation factor 1-alpha	I1GMP2	25	49.23
Elongation factor 1-alpha-like protein (Fragment)	D3G8A2	Translation elongation factor EFTu/EF1A C-terminal domain-containing protein	A0A453AJ18	7	18.70
Shoot1 protein (Fragment)	D5LXX6; I1H3B2	PDZ domain-containing protein	I1H3B2	23	36.82
Translation initiation factor 1 (Fragment)	H2B4Z9; A0A0Q3GV94; A0A0Q3HEX0; B3TN84; A8Y9C1; A0A0Q3PID2; A0A0Q3J8X1; A0A2K2CZH8	S1-like domain-containing protein	A0A0Q3GV94	2	11.75
Nonphototrophic hypocotyl 1b (Fragment)	H6BDD3	non-specific serine/threonine protein kinase[...]	A0A453BRS9	20	57.94

Cysteine protease (Fragment)	H6BDD8	Peptidase C1A papain C-terminal domain-containing protein	A0A9R0X969	10	23.89
Hypersensitive induced response protein 3 (Fragment)	H6BDE4	Band 7 domain-containing protein	I1IQE1	20	31.56
L24 ribosomal protein (Fragment)	H6BDF1; I1I840; I1HSS3	TRASH domain-containing protein	I1I840	5	18.36
Putative monodehydroascorbate reductase (Fragment)	H6BDG0	monodehydroascorbate reductase (NADH)	F2D5M0	27	46.35
Aerobic Mg-protoporphyrin IX monomethyl ester cyclase (Fragment)	H6BDG8; I1HEK1	magnesium-protoporphyrin IX monomethyl ester (oxidative) cyclase	I1HEK1	23	47.55
Glycine decarboxylase P subunit (Fragment)	H6BDH8	Glycine cleavage system P protein	A0A8R7TWG4	43	111.07
Aci-reductone-dioxygenase-like protein (Fragment)	H6BDH9; I1GTE3	Acireductone dioxygenase	I1GTE3	15	23.58
Aconitate hydratase 2 (Fragment)	H6BDI3	Aconitate hydratase	A0A9R0YB56	37	86.98
Phospoenolpyruvate carboxylase (Fragment)	H6BDI9	Phosphoenolpyruvate carboxylase	A0A453KLB7	35	65.24
Putative root border cell-specific protein (Fragment)	H6BDJ0; I1GT57; I1GT58	DUF2470 domain-containing protein	I1GT57	17	37.04
Methionine synthase 1 (Fragment)	H6BDK0	Cobalamin-independent methionine synthase MetE N-terminal domain-containing protein	A0A9R1AGF0	20	40.38
Plastid-specific 30S ribosomal protein 2 (Fragment)	H6BDL6;I1J161	RRM domain-containing protein	I1J161	16	31.5
Putative Vacuolar ATP synthase subunit d (Fragment)	H6BDL9;I1HNY4	V-type proton ATPase subunit	I1HNY4	16	40.73
glycine hydroxymethyltransferase (Fragment)	H6BDM4	Serine hydroxymethyltransferase	A0A453HCG3	17	39.75
Putative 4-hydroxy-3-methylbut-2-enyl diphosphate reductase (Fragment)	H6BDN1	4-hydroxy-3-methylbut-2-enyl diphosphate reductase	A0A453HAX1	15	34.16
Putative dehydroascorbate reductase (Fragment)	H6BDN5;I1GZH6	glutathione dehydrogenase (ascorbate)	I1GZH6	13	28.86
Protein disulfide isomerase (Fragment)	H6BDN7	Thioredoxin domain-containing protein	A0A453HK30	14	26.05
Elongation factor 2 (Fragment)	H6BDP1	Elongation factor 2	A0A9R0SNW7	18	40.18
Phosphoenolpyruvate carboxylase (Fragment)	H6BDP7	Phosphoenolpyruvate carboxylase	A0A453KLB7	35	65.24
Victorin binding protein (Fragment)	H6BDQ0	Glycine cleavage system P protein	A0A8R7TWG4	43	111.07
Beta-glucosidase 31 (Fragment)	H6BDQ4	Uncharacterized protein; 4-hydroxy-7-methoxy-3-oxo-3,4-dihydro-2H-1,4-benzoxazin-2-yl glucosidebeta-D-glucosidase	A0A453LA72; A0A8R7QGH6	15	20.48
Putative amino acid permease family protein (Fragment)	H6BDR3	Cationic amino acid transporter C-terminal domain-containing protein	A0A453JJQ2	8	29.82

Putative Xaa-Pro dipeptidase (Fragment)	H6BDR9	Peptidase M24 domain-containing protein	A0A453NTR8	6	20.99
Methylcrotonoyl-CoA carboxylase subunit alpha (Fragment)	H6BDS9; I1IGN6	Methylcrotonoyl-CoA carboxylase subunit alpha, mitochondrial	I1IGN6	37	80.8
Translation initiation factor IF- 2 domain-containing protein (Fragment)	H6WQH3	Translation initiation factor IF- 2 domain-containing protein	A0A453RVI6	11	32.62
Ribulose bisphosphate carboxylase small subunit (Fragment)	Q66LL4	Ribulose bisphosphate carboxylase small subunit, chloroplastic	A0A453AGD9	9	13.71
20S proteasome subunit alpha 3 (Fragment)	Q94KH7	Proteasome subunit alpha type	A0A0E0HLP9	11	27.04
Histone H2B-2 (Fragment)	Q94KK2; Q94KK1; Q94KJ9	Histone H2B	I1GND5	7	16.59
Photosystem II protein D1 (Fragment)	Q95GL2; A8Y9F0; A0A2K2CNR4; A0A2K2CGK7	Photosystem II protein D1	A8Y9F0	10	38.93
L3 ribosomal protein (Fragment)	Q9AT58	60S ribosomal protein L3	A0A453HMB0	15	24.84

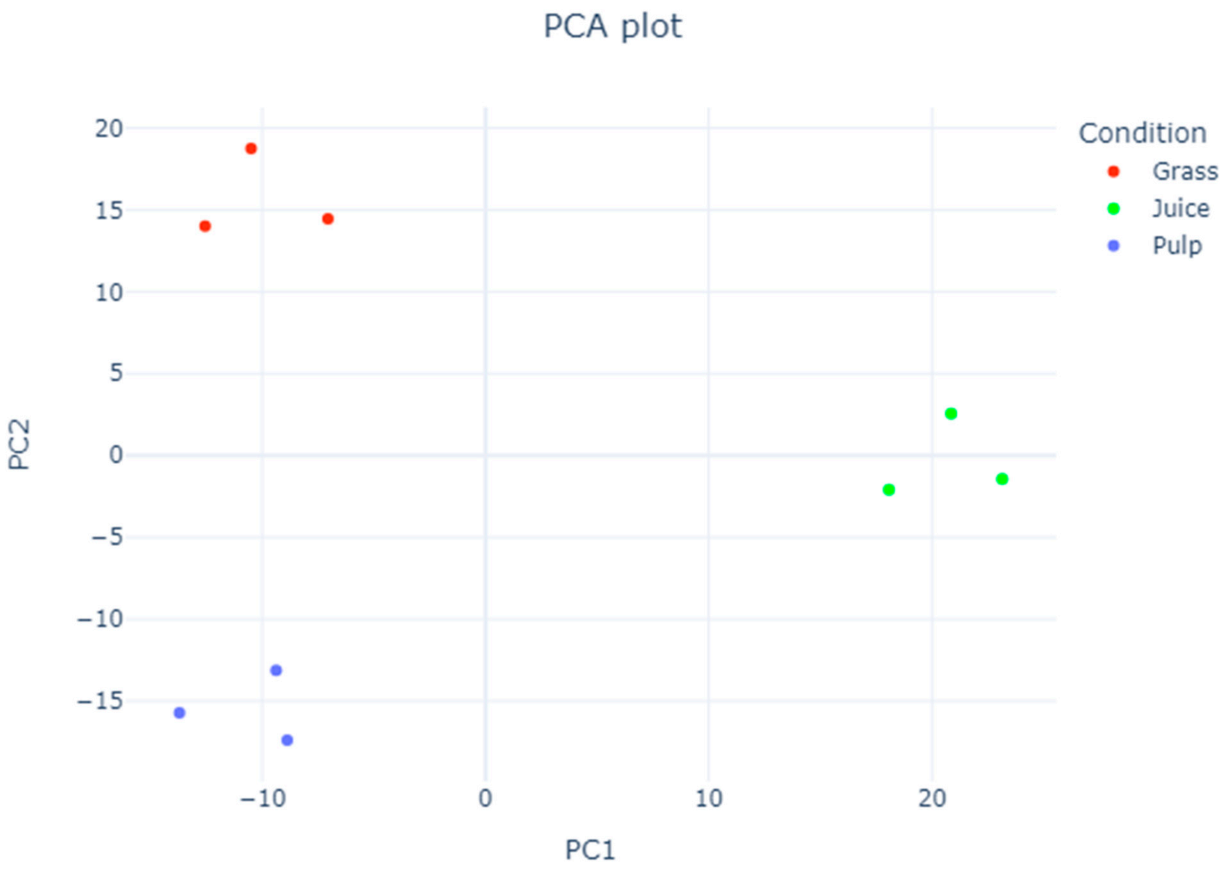

Figure S1: PCA plot triplicate analysis of initial crude fractions. From Scree plot analysis (data not shown), PC1 explains 20.2% of the data variability while PC2 explains 15.3%.

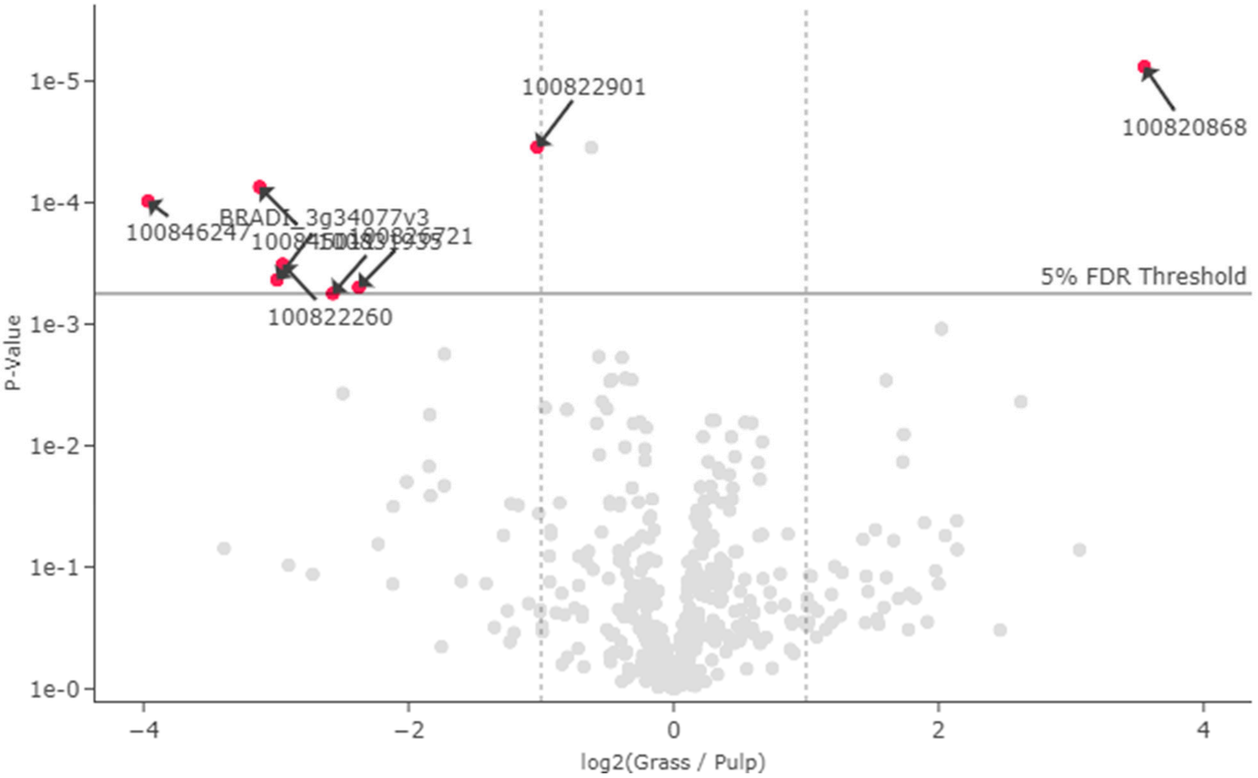

Figure S2: Volcano plot of differentially abundant proteins in pair-wise comparison of grass and pulp. Differentially abundant proteins (log2 fold change > 1, adjusted p-value < 0.05 (5% FDR threshold)) are indicated by arrows and red dots and annotated by gene name (see Table S1).

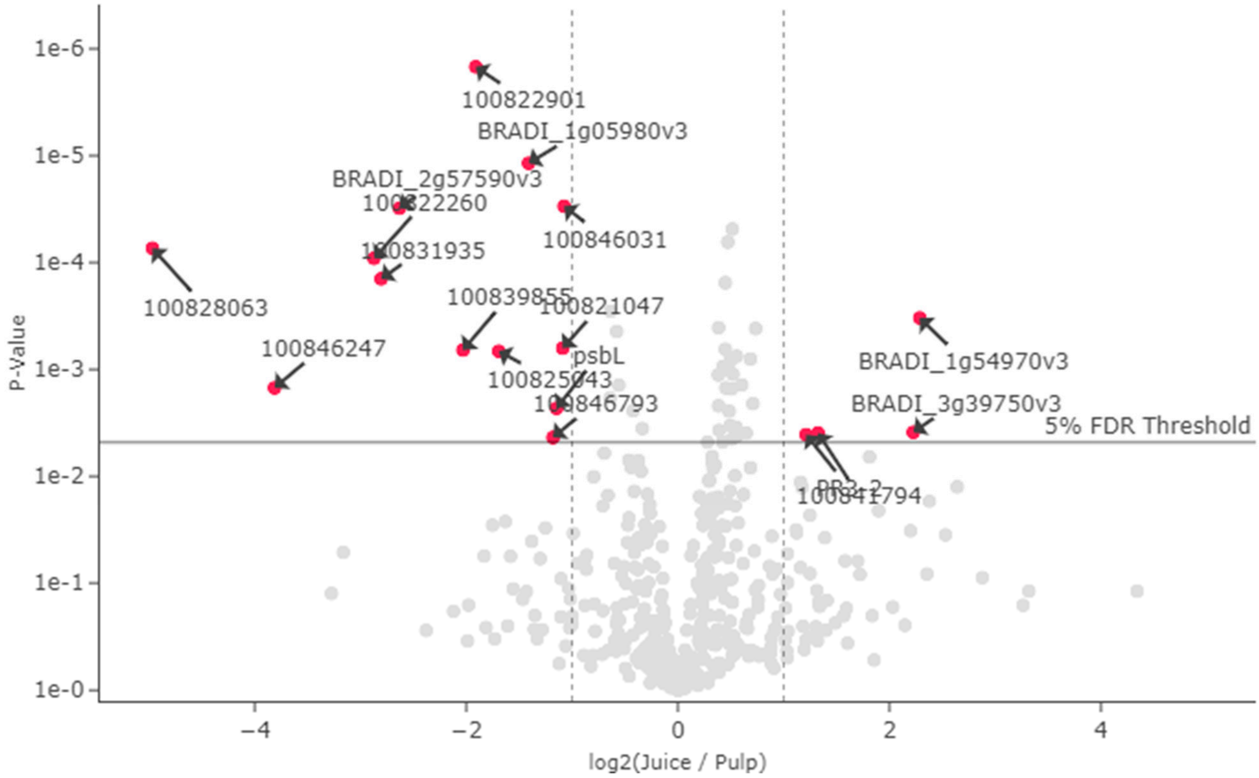

Figure S3: Volcano plot of differentially abundant proteins in pair-wise comparison of juice and pulp. Differentially abundant proteins (log2 fold change > 1, adjusted p-value < 0.05 (5% FDR threshold)) are indicated by arrows and red dots and annotated by gene name (see Table S1).

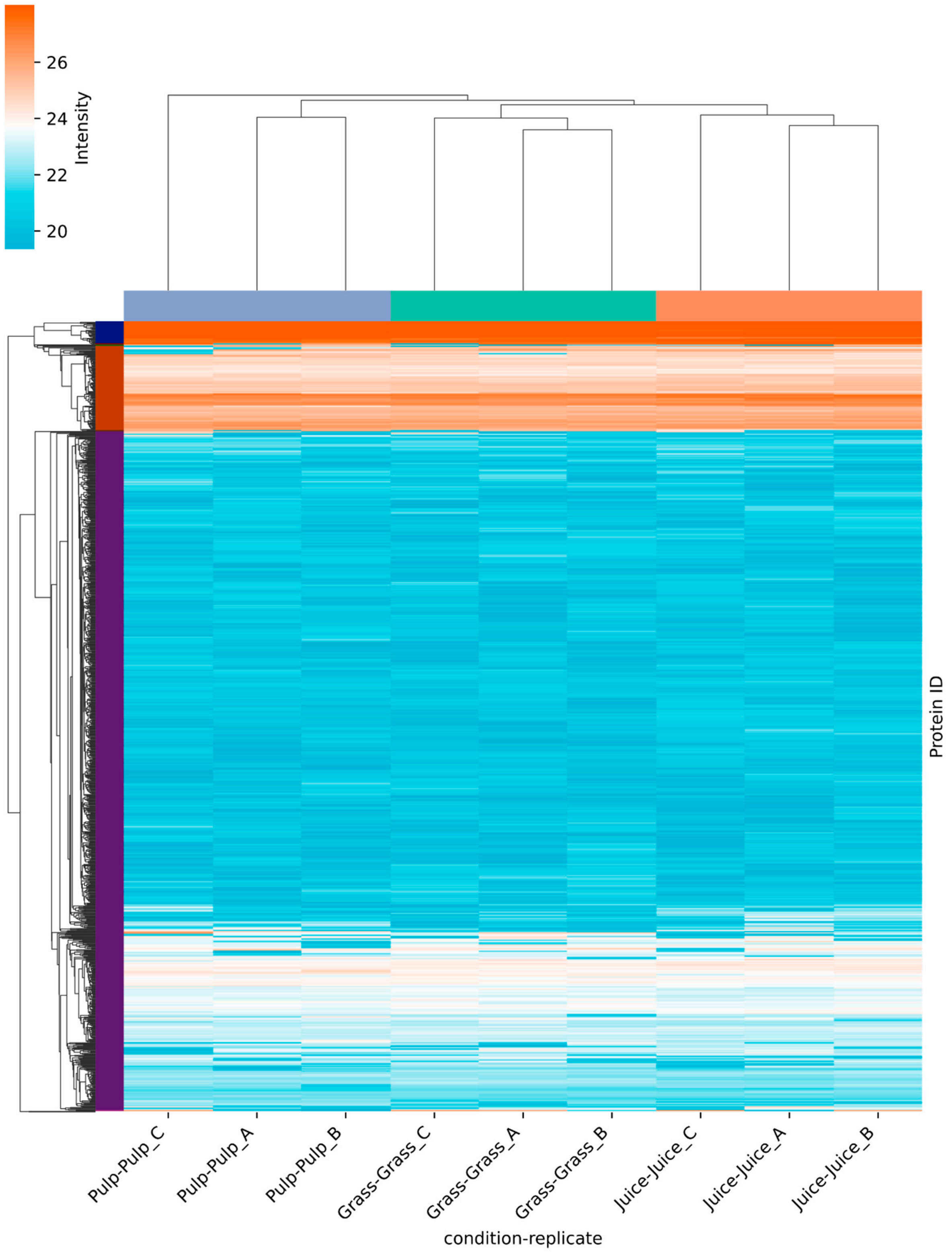

Figure S4: Heatmap representation of all 1084 identified proteins (rows) across triplicate analysis of all three crude fractions. In the heatmap, LFQ intensities are shown without standardized (z-score normalization) proteins are clustered by similarity using a cluster distance of ten. Eight clusters are observed containing 31 proteins (blue), 117 protein (brown), 931 proteins (purple) as well as five clusters containing a single protein. Crude fractions and replicates (columns) are clustered hierarchically.

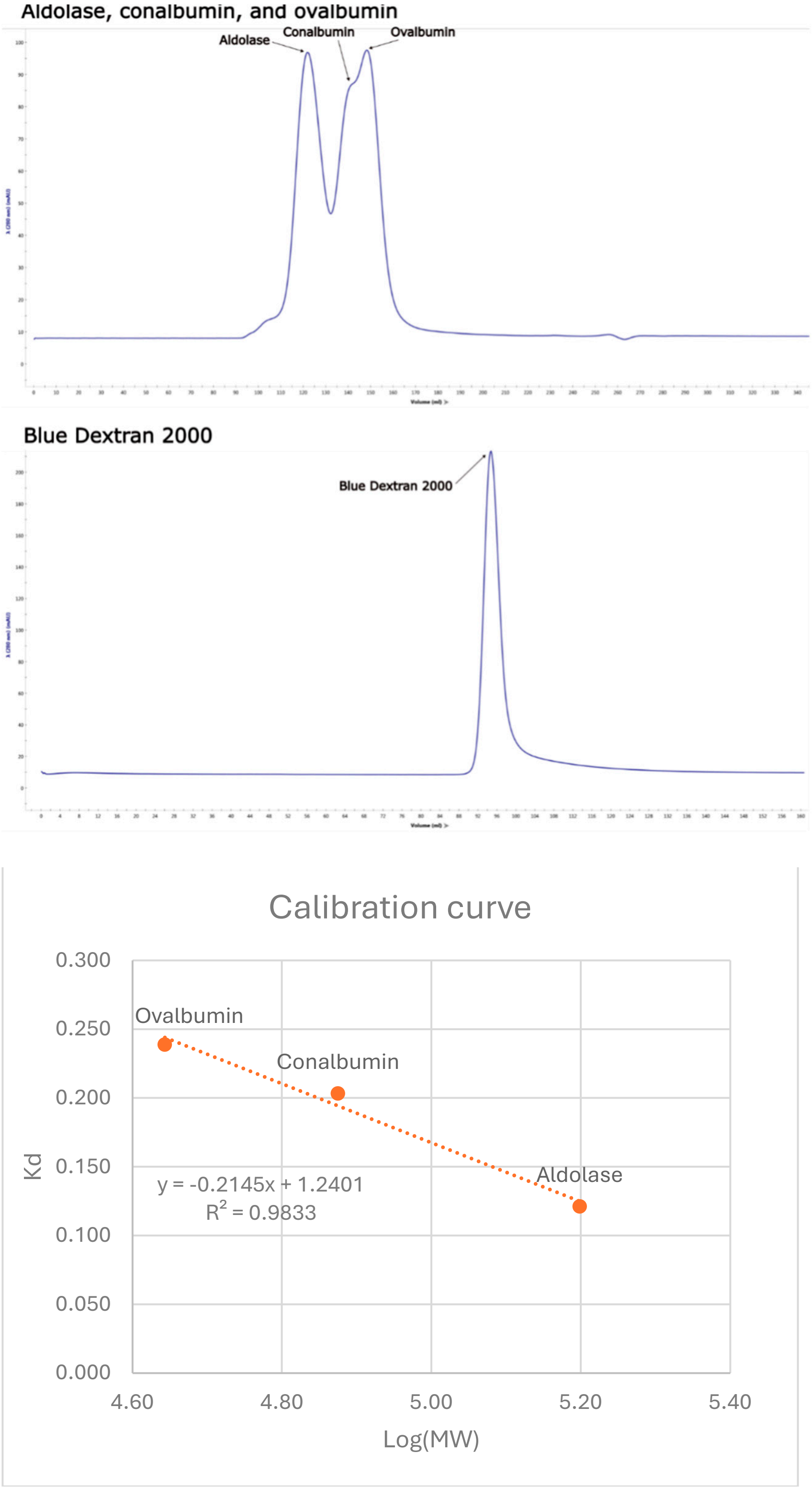

Figure S5: Chromatogram and calibration curve of standards using 320 mL Sephacryl column. Top) Elution profiles of calibration mix and Blue Dextran 2000 in PBS buffer. Bottom) Calibration curve of standards based on distribution coefficients (Kd) as function of logarithmic molecular weights (log(MW)).

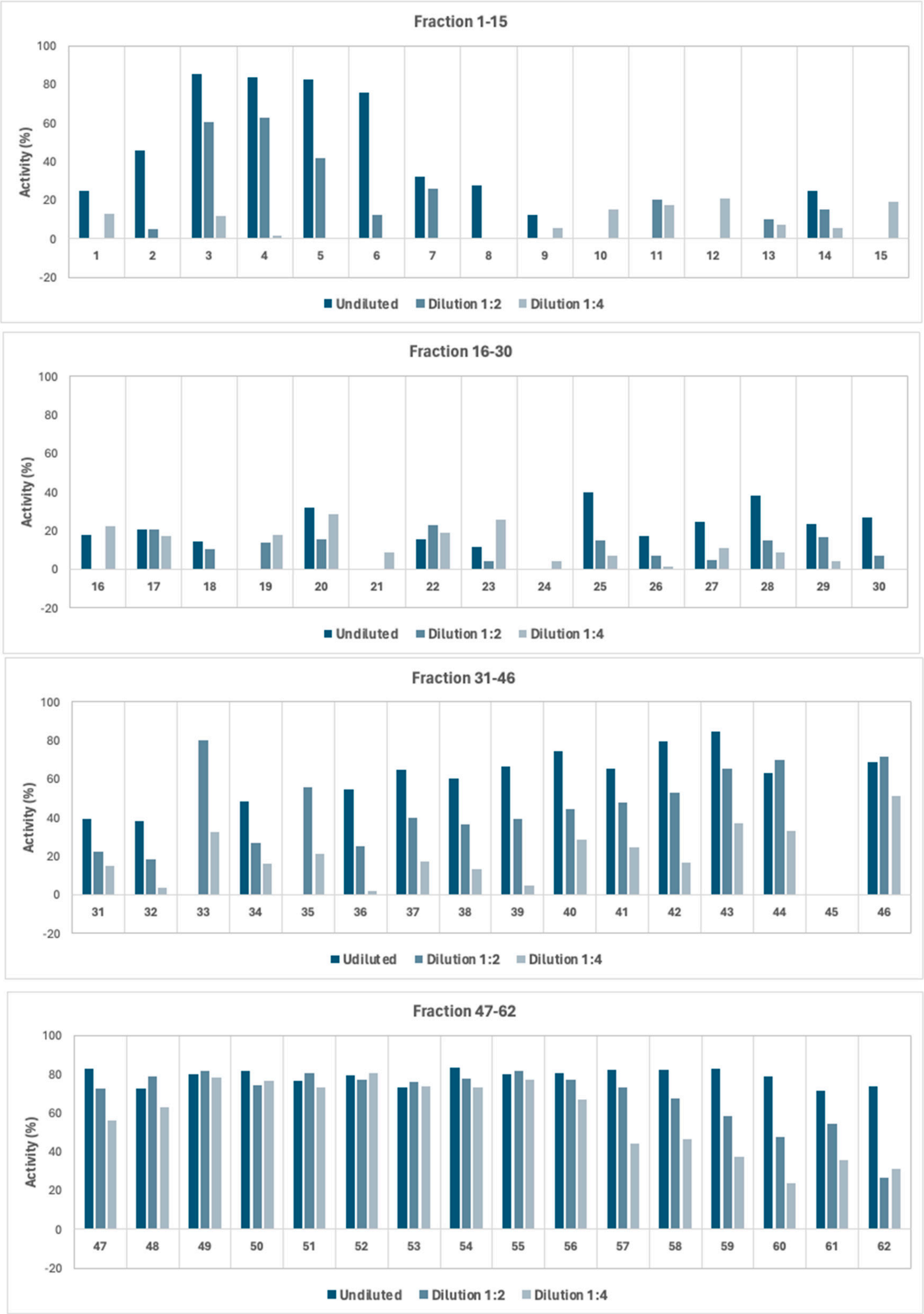

Figure S6: Activity of all fractions for each dilution in the DPPH radical scavenging screening. Certain data points are excluded due to negative activity values.

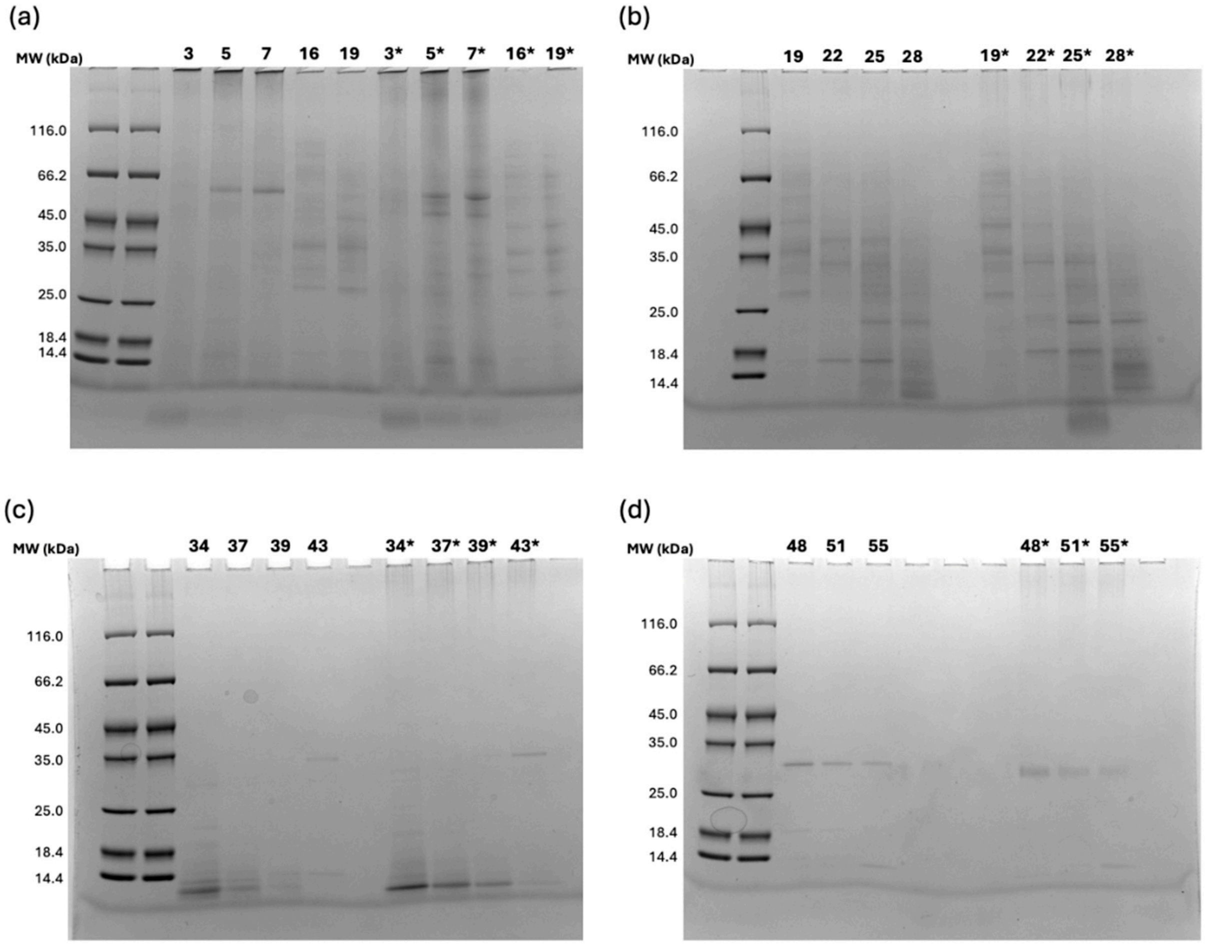

Figure S7: Reducing (*) and non-reducing SDS-PAGE analysis of the 15 selected fractions.

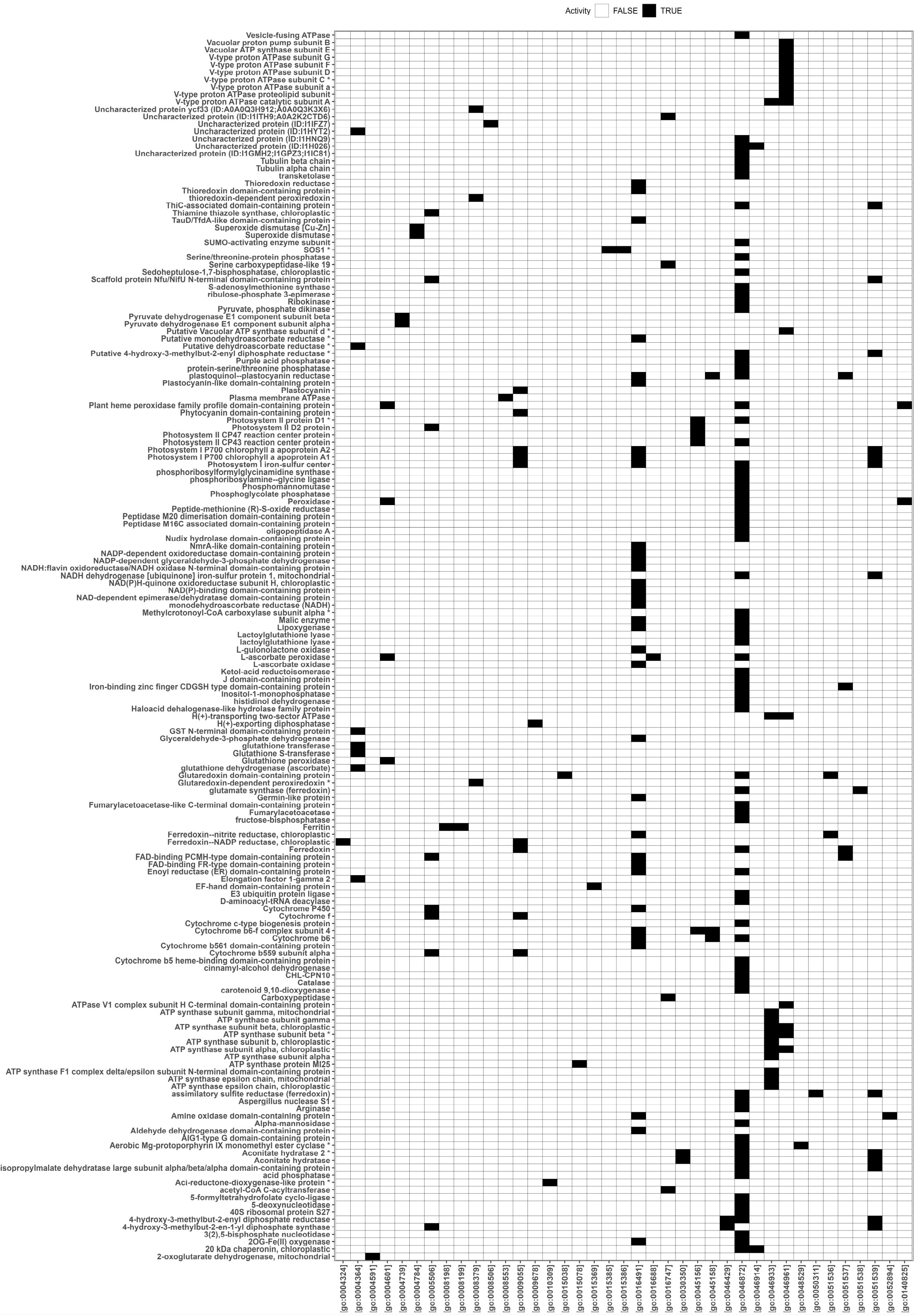

Figure S8: Qualitative analysis of GO-annotated proteins. The analysis was used to shortlist potential antioxidant proteins in the protein dataset for SEC fractions, yielding 166 candidates (representing 235 protein groups) for further investigation, and subsequent quantification in the crude fraction dataset. Go-terms are indicated by term ID to improve data overview.
